# Exploring infectious aneurysms through a series of cases in a tertiary care unit in South India

**DOI:** 10.12688/f1000research.171966.1

**Published:** 2025-12-03

**Authors:** Keerthiraj B, Bergin C, Pooja Rao, Udayalaxmi Jeppu

**Affiliations:** 1 Kasturba Medical College Mangalore, Manipal Academy of Higher Education, Manipal, India

**Keywords:** Mycotic aneurysm; pulmonary artery aneurysm; post infectious aneurysm; case series aneurysm

## Abstract

**Background and aims:**

Infectious aneurysm is an aneurysm of the arteries originating from endogenous bacteraemia due to bacterial or fungal origin. The major vessels involved are femoral, aorta, then intracranial visceral arteries and pulmonary arteries. These aneurysms are identified by their fusiform shape, multiple, distal location involving the entire wall of the vessel and changing morphology on repeat imaging studies. The study aims to discuss the clinical presentation & management of post infectious aneurysms.

**Methods:**

This is a retrospective study of 11 case series of postinfectious aneurysms over a period of 10 years admitted at a tertiary care hospital. The detailed clinical data that included the clinical presentation, diagnostic and treatment was studied from the hospital medical records department and laboratory information system.

**Results:**

Among the 11 patients diagnosed with aneurysms, 4 presented with intracranial, 4 with intra pulmonary and 3 with intra-abdominal aortic aneurysms involvement. Source was either of bacterial cause such as *E.coli, Streptococcus gallolyticus, Salmonella* Typhi, *M. tuberculosis, Klebsiella pneumoniae*, Methicillin Resistant *S. aureus, S. hominis* or fungal origin being *Aspergillus spp.* All of them had elevated C-reactive protein level, ESR and confirmed by positive cultures or Xpert Tb. Management of mycotic aneurysm includes imaging studies along with laboratory workup, endovascular intervention with intravenous antibiotics, or a combination of these.

**Conclusion:**

High index of suspicion in patients presenting with fever, elevated C-reactive protein, ESR, total counts with sudden development of atypical aneurysms requires timely blood culture pre and post procedure. Definitive surgical/endovascular under antibiotic coverage at the earliest is a prerequisite in the management to prevent re-rupture.

## Introduction

Infectious aneurysms are a rare type of intracranial aneurysm distinguished from the more common berry aneurysms by their infectious origin, occurrence in younger individuals, multiple occurrences, and distal aneurysm locations. The key histopathological feature of these aneurysms is infection and arterial wall destruction. ^1^ Consequently, they are pseudoaneurysms that can arise from infections spread through endovascular means (such as infective endocarditis) or extravascular means (such as meningitis). The term “infectious aneurysm” is likely more appropriate than MA, as these aneurysms can be caused by various bacteria, fungi, mycobacteria, and viruses. The most frequently involved organisms are *Streptococcus viridans* and *Staphylococcus aureus.* It most commonly presents as intracranial hemorrhage, and, compared with other aneurysms, it has a greater risk of rupture and fatal bleeding. ^2^

## Materials and methods

An analytical, cross-sectional study was conducted over a 10-year period from 2013 2023 at a tertiary care centre in Southern India. The study was approved by the Institutional Ethics Committee (IEC) of Kasturba Medical College, Mangalore with Reg no. IEC KMC MLR 07/2024/476. In compliance with the Declaration of Helsinki, the IEC granted the patient consent waiver to review the medical records where the participants could not be contacted, and there was absolutely no risk to the patient, with all the data being anonymized. Symptomatic patients admitted to the hospital with a diagnosis of postinfectious aneurysm by imaging studies were considered during the study period. The ethics committee waived the need to obtain informed consent, as the data were obtained from the laboratory information system and hospital medical records department of the attached hospitals, and the tests and procedures performed were part of the investigations ordered for the patient during the routine course of diagnosis and treatment. The inclusion criterion was symptomatic patients admitted to the hospital and diagnosed with postinfectious aneurysms during the investigation workup. The exclusion criterion was patients with aneurysms of noninfectious origin. The clinical and laboratory parameters, imaging studies and management of the patients, as well as their outcomes, such as recovery/death, were captured via Excel software. The data were analysed with the help of frequencies and proportions for categorical values, whereas the quantitative data were expressed as means, medians, and proportions.

## Results

Eleven cases were considered via the convenience sampling method during the study period. Four patients presented with intracranial aneurysms, 4 with intrapulmonary aneurysms and 3 with intraabdominal aortic aneurysms ( Table 1). The median age of these patients was 58 years, and these patients were more common among males than females ( Table 2).

**Table 1.** Summary of 11 cases with infectious aneurysms.

Case	Aneurysm number	Location	Shape	Size	Diagnosis	Procedure
1	1	Abdominal aorta	Saccular	26 × 15 mm	Abdominal aortic aneurysm	DSA aortogram and endovascular covered stent placement
2	1	Abdominal aorta	NA	NA	Abdominal aortic active leak with retro peritoneal haematoma	Aortogram and endovascular aortic stent graft placement along with inj. Teicoplanin followed by Linezolid
3	1	Abdominal aorta	Saccular	5 × 5.6 cms	Infrarenal abdominal aortic aneurysm	Endovascular aortic stent graft placement for infrarenal abdominal aortic aneurysm with partial thrombus and contained rupture along with injection ceftriaxone
4	2	Left & right ACA	Fuso saccular	2.7 × 2.6 mm & 2.8 × 2.5 mm	Stroke, left parieto occipital lobe bleed, B/L distal Ant cerebral artery	Endovascular glue embolization with parent artery occlusion of distal pericallosal artery branch of left ACA and distal inferior parietal branch of right ACA post infectious
5	30-35 (multiple)	ICA, MCA, ACA, PICA	Fusiform	NA	Intracranial bleed, aneurysm rupture with cutaneous Nocardiosis	DSA and I. V Meropenem & tab cotrimoxazole
6	1	MCA	Fuso saccular	11 × 22 mm	Intraparenchymal haemorrhage (left parietal of MCA aneurysm)	Endovascular glue embolization of parietal branch of left MCA with parent artery occlusion for left MCA parietal branch
7	1	Right Distal ACA	Irregular saccular	3.9 × 3.6 mm	SAH with ruptured intracranial aneurysm	DSA cerebral angiogram, endovascular coiling (coil trapping) of ruptured right DACA irregular saccular aneurysm with intravenous Meropenem
8	1	Right Pulmonary artery	NA	2 × 2.2 × 2.5 cm	Pulmonary artery pseudoaneurysm	PVA particles embolization of right bronchial artery and CT guided percutaneous glue injection for left upper lobar apico-posterior segment pulmonary artery pseudoaneurysm
9	1	Right Pulmonary artery branch aneurysm	NA	1 × 1 cm	Rasmussen aneurysm	Percutaneous glue embolization of right upper lobe posterior segment pulmonary artery branch aneurysm (Rasmussen aneurysm) with complete obliteration of the pseudoaneurysm from the circulation with no active leak and normal filling of rest of the right pulmonary artery
10	1	Right pulmonary artery	NA	7.5 × 8 cm	Pulmonary artery pseudoaneurysm	Endovascular amplatz vascular plug, coil, glue embolization of large right lower lobe pulmonary artery aneurysm with complete obliteration of aneurysm and persisting flow in right upper lobe pulmonary artery
11	1	Right pulmonary artery	NA	4 × 1.4 × 2.3 cms	Large pulmonary artery aneurysm	DSA & CT guided glue and coil embolization of partially thrombosed large pulmonary artery aneurysm

**Table 2.** Clinical profile of 11 cases presenting with infectious aneurysm.

Case	Clinical presentation	Age/Gender	Organism	Comorbidities
1	Fever since 4 days, Severe lower abdominal pain	66/F	*S hominis*	Hypertension, diabetes
2	Fever, lower abdominal & flank pain since 1 week	59/M	*Methicillin Resistant Staphylococcus aureus*	Diabetes, hepato-splenomegaly, renal calculi
3	Lower abdominal pain, constipation since 1 week and hypovolemic shock	62/M	S Typhi	Hypertension, hypercholesterolemia
4	Headache since 2 months, sudden onset of left hemiplegia	58/M	*S gallolyticus*	NIL
5	Fever, vomiting, nausea generalized weakness since 1 day	62/F	*Nocardia spp*	CKD */HTN †/post kidney transplant/UTI ‡
6	Fever, vomiting with seizures	23/M	*Streptococcus mitis* (viridans group)	IE of mitral valve, Anemia
7	Headache, altered sensorium, low Glass glow Coma Scale	65/M	*Klebsiella pneumoniae*	H/O fall, Hypertension
8	Recurrent massive haemoptysis	52/M	*Mycobacterium tuberculosis*	NA
9	Recurrent haemoptysis with cavitary lesion	62/M	*Mycobacterium tuberculosis*	NA
10	Hemoptysis	65/M	*Aspergillus fumigatus*	NA
11	Massive hemoptysis	64/M	*Mycobacterium tuberculosis*	NA

Footnotes:

* Chronic Kidney Disease;

^†^ Hypertension;

^‡^ Urinary tract Infections.

**Figure 1.**
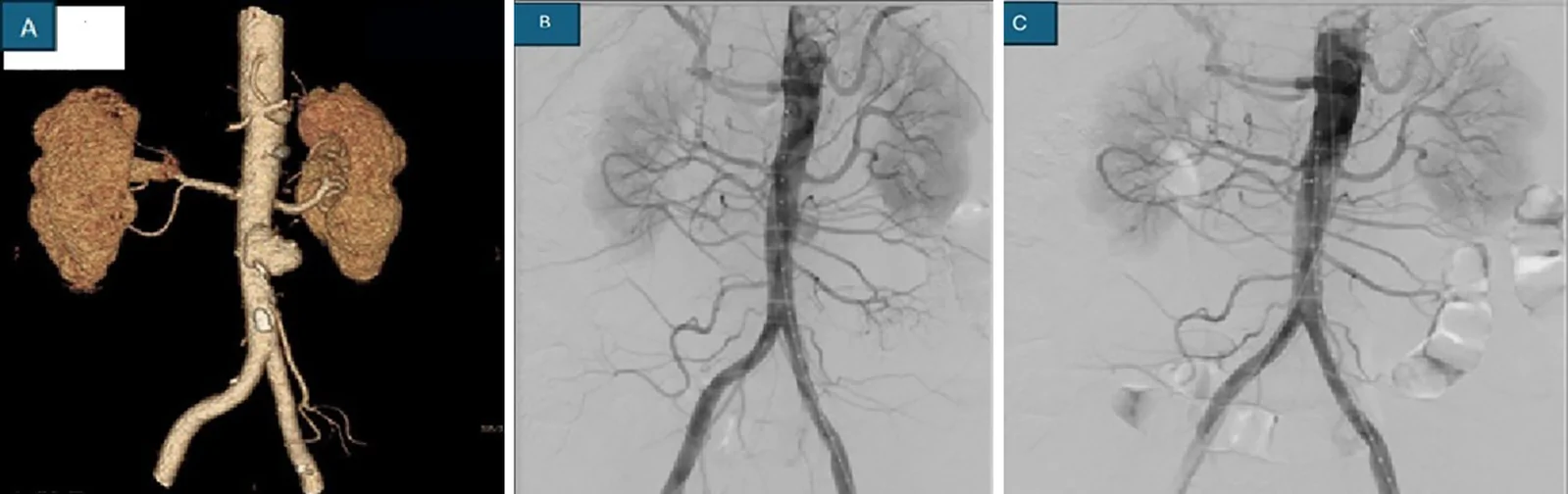
A) Pre procedure: CT angiogram of intra-abdominal aorta aneurysm. B) Pre procedure: Aortogram showing infrarenal abdominal aortic saccular aneurysm. C) Post procedure: Aortogram showing no filling of the aneurysm.

The size of the aneurysms was small (<5 mm) in 3 patients, whereas large aneurysms were noted in the remaining 6 patients. All these patients had elevated C-reactive protein levels and ESRs. No immediate postoperative complications were observed. The sources of these postinfectious aneurysms were bacterial or fungal in origin ( Table 2). The identification was based on blood culture or GeneXpert for tuberculosis or fungal culture results. CT angiogram, digital subtraction angiography (DSA) or ultrasonography are considered important diagnostic tools ( Table 1) ( Figure 1).

Management was either endovascular with coil placement or embolization along with appropriate antibiotic therapy ( Table 1). During follow-up, no patients experienced recurrence or rebleeding. One patient with cutaneous nocardiosis with multiple saccular aneurysms died because of intracranial bleeding. Endovascular intervention could not be performed because there were multiple aneurysms.

## Discussion

Sir William Osler coined the term “mycotic aneurysm” in 1885 because of its appearance as a mushroom. ^3^ The term primary infectious aneurysm is best suited for aneurysms of arteries that develop immediately because of an infectious cause. These aneurysms account for no more than 3% of all aneurysms in the West, with infectious intracranial aneurysms rarely accounting for approximately 0.7% of all aneurysms. This condition tends to occur in younger men than in those who develop degenerative aneurysms. ^4– 6^ These aneurysms grow rapidly and have high chances of rupture, with an increase in mortality of up to 60%. ^7^ The location and time of the survey impact pathogen identification in cases of mycotic thoracic aortic aneurysm (MTAA), which can vary significantly. Before the advent of antibiotics, gram-positive cocci, such as *Staphylococcus* and *Streptococcus*, were commonly implicated in endocarditis. However, in the last two decades, gram-negative organisms have emerged more prominently, accounting for up to 40% of MTAA cases. Among them, the most prevalent pathogens are *Staphylococcus spp, Streptococcus spp*, and nontyphoidal *Salmonella* species. In Western countries, *Staphylococcus aureus* (28%), *Salmonella* spp. (15%), and *Pseudomonas aeruginosa* (10%) are the most common causative organisms. In contrast to Asian countries, *Salmonella* remains the predominant pathogen, of which non typhoidal Salmonella such as *Salmonella typhimurium*, *S.* e *nteritidis* (serogroup D), and *S.* *choleraesuis* (serogroup C) are the most frequently isolated species. Additionally, other gram-positive bacteria (e.g *., Clostridium, Corynebacterium, Enterococci*), gram-negative bacteria (e.g., *E. coli, Enterobacter, Serratia, Haemophilus influenzae, Proteus vulgaris, Yersinia enterocolitica, Burkholderia pseudomallei, Klebsiella pneumoniae, Coxiella burnetii, Campylobacter*), and anaerobic organisms (e.g., *Bacteroides fragilis*), as well as less common but notable pathogens such as *Treponema pallidum* and *Mycobacterium tuberculosis*, may contribute to the pathogenesis of MTAA. ^8^ The semiautomated Vitek 2 system contributes to approximately 50% to 85% of the diagnoses of etiology from blood cultures, which are positive in patients with a mycotic aneurysm. Majority of cases typically involve a single organism that is isolated, although multiple organisms can be found in 8% of cases, followed by 25% of cases where the etiology is unknown. The most identified pathogens include nontyphoid *Salmonella, Staphylococcus, Campylobacter,* and *Streptococcus,* but *E. coli, Mycobacteria,* and Bacteroides species have also been reported. Identifying infective microorganisms and confirming their antibiotic sensitivity can aid in the administration of targeted antimicrobial selection. However, bacterial isolation may be challenging due to culture difficulties or prior antibiotic treatment. Molecular methods such as multiplex polymerase chain reaction (PCR) can improve organism identification from blood cultures. ^3^

A study by Alawieh et al reported that 44.2% of IIAs were small in size (<5 mm), 56.9% were traced in the MCA, and 13.1% were traced in the PCA. ^9^ Prompt recognition of these cases is an effective tool for the management of postinfectious aneurysms. Timely diagnosis of a mycotic TAAA requires a high index of suspicion in patients presenting with fever, headache, back pain, pain abdomen, or a pulsatile abdominal mass. ^4, 10^ In our study, TAAA patients presented with similar symptoms, namely, acute onset of fever and abdominal pain followed by hypovolemic shock. Although leucocytosis and elevated erythrocyte sedimentation rates are common, these findings are nonspecific and can often lead to delayed or incorrect diagnoses. Many a times blood cultures may be negative for bacteraemia, especially if there is before antibiotic administration. Contrast-enhanced computed tomography can suggest infective features, such as an atypical setting of the aneurysm; a multilobulated, multifocal or saccular configuration; lacking calcification in the aneurysmal wall; gas in the periaortic area with a soft tissue reaction; and adjacent vertebral osteomyelitis. In uncertain cases, Gallium-67 isotope scanning may help in localizing the active infectious process and with a hold on septic foci at other site. Angiography, when feasible, is useful tool for defining the anatomy of the visceral vessels and aiding in preoperative planning. Even if noninvasive tests are negative, DSA remains the yardstick and is recommended for the detection of IIAs. ^11, 12^

Some patients in our series were initially treated for conditions such as acute pyelonephritis, presenting typically with fever, flank pain, and on kidney echography, or spinal osteomyelitis showing hydronephrosis, presenting with fever, malaise, and back pain in bedridden patients before the right diagnosis of an infectious aneurysm was made. ^13^

In patients with ruptured IIAs and intra-abdominal aneurysms or in those who fail conservative therapy, endovascular intervention is the mainstay in comparison with microsurgical craniotomies as it offers significantly lower mortality, better accessibility to distal aneurysms, superior outcomes in the treatment of multiple aneurysms, patients with comorbidities, and a decreased risk of anticoagulation. ^9^ Endovascular intervention was performed with stent graft placement for intra-abdominal aneurysms. Embolization with liquid agents such as glue and/or coils was considered for pulmonary artery or intracranial aneurysms. The placement of coils to obliterate the aneurysm was also considered for intracranial aneurysms. The role of surgical intervention should be considered in cases involving large intraparenchymal hematomas or significant cerebral edema or when revascularization is needed. Administration of intravenous post op antibiotics in the hospital for at least 4 weeks guided by clinical and laboratory parameter normalization. ^7^

## Conclusion

Mycotic aneurysms (MAs) present significant clinical challenges because of their rare occurrence and the complexities involved in their diagnosis and management. A high index of suspicion for MAs should be maintained in patients with fever, elevated inflammatory markers such as C-reactive protein and ESR, and sudden development of atypical aneurysms. Prompt blood culture and appropriate imaging are crucial for timely diagnosis. Effective management also includes a comprehensive laboratory workup and imaging studies to guide treatment decisions and monitor progress.

The management of MAs requires a multifaceted approach that combines surgical and medical interventions. Surgical options include clipping or clamping with reconstruction and graft placement, whereas endovascular treatments are reserved for hemodynamically unstable or medically fragile patients. Postoperative care involves prolonged antibiotic therapy to ensure infection resolution and prevent recurrence. Ultimately, this study underscores the necessity of early and definitive surgical or endovascular intervention under antibiotic coverage to improve outcomes and reduce the risk of aneurysm re-rupture.

## Data Availability

The authors confirm that the data supporting the findings of this study are available within the article.
