# Supplementary material for: Exploring infectious aneurysms through a series of cases in a tertiary care unit in South India
Source: F1000Res. 2025 Dec 3;14:1356. [Version 1] doi: 10.12688/f1000research.171966.1 (PMC13560017; doi:10.12688/f1000research.171966.1)
Supplement: Peer review history

# Reviewer report: Reviewer response for version 1

**DOI:** 10.5256/f1000research.189639.r497554

**Published:** 2026-08-18

**Authors:** Rama Shankar Rath

**Affiliations:** 1 All India Institute of Medical Sciences, Gorakhpur, Uttar Pradesh, India

Thank you for providing me the opportunity to review this manuscript. It is a nicely written and well-drafted manuscript on a very important topic. This article will be helpful for clinicians, microbiologists, and public health experts working in the field of mycotic aneurysms. Since this is a case series, there are no methodological issues. The authors have clearly conducted all the required investigations as part of appropriate patient care and have described them comprehensively in the manuscript. The methods used were appropriate for the study, and the results are clearly presented. The tables and figures are relevant, informative, and appropriately support the findings.

Is the work clearly and accurately presented and does it cite the current literature?

Yes

If applicable, is the statistical analysis and its interpretation appropriate?

Not applicable

Are all the source data underlying the results available to ensure full reproducibility?

Yes

Is the study design appropriate and is the work technically sound?

Yes

Are the conclusions drawn adequately supported by the results?

Yes

Are sufficient details of methods and analysis provided to allow replication by others?

Yes

Reviewer Expertise:

ygoloimedipE

I confirm that I have read this submission and believe that I have an appropriate level of expertise to confirm that it is of an acceptable scientific standard.

# Reviewer report: Reviewer response for version 1

**DOI:** 10.5256/f1000research.189639.r439175

**Published:** 2025-12-30

**Authors:** Richa Sinha

**Affiliations:** 1 Microbiology, Dr.Ram Manohar Lohia Institute of Medical Sciences, Lucknow, Lucknow, Uttar Pradesh, India; 2 Sanjay Gandhi Post Graduate Institute of Medical Sciences, Lucknow, Uttar Pradesh, India

This article provides crucial, region-specific data on infectious aneurysm highlighting their diverse causes (like *Salmonella*, *E. coli*, fungi) in Indian patients.

 The author have well mentioned the details of 11 retrospective cases of infectious aneurysms (IA), and have highlighted high mortality in high-risk patients (hypertension, diabetes). This article gives emphasises on open surgery over EVAR for better infection clearance, and recommends post-surgery IV antibiotics to prevent recurrence.

Is the work clearly and accurately presented and does it cite the current literature?

Yes

If applicable, is the statistical analysis and its interpretation appropriate?

Yes

Are all the source data underlying the results available to ensure full reproducibility?

Yes

Is the study design appropriate and is the work technically sound?

Yes

Are the conclusions drawn adequately supported by the results?

Yes

Are sufficient details of methods and analysis provided to allow replication by others?

Yes

Reviewer Expertise:

Infectious disease , Microbiology

I confirm that I have read this submission and believe that I have an appropriate level of expertise to confirm that it is of an acceptable scientific standard.
